# Sustainable nutrition and athlete well-being: associations with psychological and physical performance indicators

**DOI:** 10.3389/fnut.2026.1871130

**Published:** 2026-06-26

**Authors:** Zhiyong Li, Yang Yu

**Affiliations:** 1 Sports Training College of Guangzhou Sport University, Guangzhou City, Guangdong, China; 2 School of Sports and Health, Guangzhou Sport University, Guangzhou City, Guangdong, China

**Keywords:** diet, physical outcomes, psychology, sports, sustainable food

## Abstract

**Background:**

Sustainable dietary practices are increasingly recognized for their potential relevance to both environmental sustainability and athlete health. However, limited research has examined the relationship between sustainable nutrition behaviors and psychological and physical performance indicators among athletes.

**Objective:**

This study aimed to examine the associations between sustainable food and diet choices, sports psychology outcomes, and perceived physical performance indicators among physically active individuals.

**Methods:**

A quantitative cross-sectional study was conducted among 250 physically active participants recruited from sports clubs, university teams, fitness centers, and training groups. Data were collected using a structured questionnaire assessing sustainable dietary practices, psychological outcomes (motivation, mental focus, mood stability, and psychological readiness), and perceived physical outcomes (energy level, recovery quality, fatigue control, and perceived performance). Descriptive statistics, Pearson correlation analysis, and multiple regression analysis were performed to evaluate associations among study variables.

**Results:**

Participants reported moderate-to-high levels of sustainable dietary practices (mean = 3.76 ± 0.61). Sustainable diet choices were positively associated with sports psychology outcomes (*r* = 0.52, *p* < 0.01) and physical outcomes (*r* = 0.48, *p* < 0.01). Regression analysis indicated that sustainable dietary practices were significant predictors of sports psychology outcomes (*β* = 0.38, *p* < 0.001) and perceived physical outcomes (*β* = 0.35, *p* < 0.001), after adjusting for training frequency, competition level, age, and gender.

**Conclusion:**

The findings suggest that sustainable dietary practices are associated with more favorable psychological well-being and perceived physical performance indicators among athletes. Although causal relationships cannot be established due to the cross-sectional design, the results highlight the potential relevance of sustainable nutrition within athlete health and performance contexts. Future longitudinal and experimental studies are needed to confirm these relationships and examine underlying mechanisms.

## 1 Introduction

Nutrition plays a fundamental role in athletic training, recovery, health, and performance. In sports science, dietary intake has traditionally been examined in relation to physiological outcomes such as energy availability, muscle recovery, body composition, endurance, and strength performance (1). Adequate intake of carbohydrates, proteins, fats, micronutrients, and fluids is essential for maintaining training adaptation, immune function, and recovery processes in athletes. Insufficient or unbalanced nutritional intake may negatively affect performance through fatigue, impaired recovery, reduced concentration, and increased injury risk.

Beyond physical performance, nutrition is increasingly recognized as an important factor influencing psychological well-being in sport. Sports psychology research demonstrates that athletic performance is strongly affected by motivation, emotional regulation, cognitive focus, stress management, confidence, and psychological readiness. Nutritional status may contribute to these factors through mechanisms related to energy balance, blood glucose regulation, micronutrient adequacy, sleep quality, inflammation control, and overall well-being (2). In particular, inadequate dietary intake and low energy availability have been associated with fatigue, mood disturbance, reduced concentration, and impaired recovery among athletes. The International Olympic Committee consensus statement on Relative Energy Deficiency in Sport (RED-S) further emphasizes that insufficient energy intake may adversely affect both physiological and psychological functioning in athletes (3).

At the same time, growing global attention has been directed toward sustainable dietary practices due to concerns regarding environmental sustainability, food systems, and long-term public health. Sustainable diets are generally characterized by nutrient-dense foods, reduced reliance on highly processed products, lower environmental impact, reduced food waste, and balanced consumption patterns that support both human and environmental health (4). The Food and Agriculture Organization and the World Health Organization define sustainable healthy diets as dietary patterns that promote health and well-being while remaining environmentally sustainable, culturally acceptable, accessible, and affordable (5).

In sports nutrition, interest in sustainable dietary practices has increased considerably in recent years. This is partly because athletes often consume large quantities of food and nutritional supplements, potentially increasing the environmental impact of dietary behaviors. Sustainable sports nutrition strategies may include greater consumption of fruits, vegetables, legumes, whole grains, nuts, seeds, and minimally processed foods, alongside reduced food waste and more efficient meal planning (6). Emerging evidence suggests that appropriately planned sustainable dietary patterns, including plant-based or flexitarian approaches, may adequately support athletic performance when energy and nutrient requirements are met (7).

Several studies have examined the physiological effects of diet quality on athletic performance, recovery, and training adaptation. Nutrient-dense dietary patterns rich in antioxidants, fiber, vitamins, minerals, and healthy fats may support muscle recovery, immune function, and exercise adaptation (8). In addition, diets emphasizing minimally processed foods may contribute to improved energy stability and reduced fatigue. However, sustainable dietary approaches in athletes remain an area of debate because some athletes and coaches continue to express concerns regarding protein quality, iron intake, vitamin B12, omega-3 fatty acids, and overall energy adequacy in more sustainability-oriented diets (9). Consequently, sustainable sports nutrition requires careful planning to ensure that performance and recovery demands are met.

Despite growing interest in sustainable nutrition, empirical research examining its relationship with athlete psychological well-being and perceived physical performance remains limited. Existing literature has largely focused either on environmental sustainability or on traditional sports nutrition outcomes independently. Comparatively few studies have simultaneously investigated sustainable dietary practices alongside psychological outcomes such as motivation, mental focus, mood stability, and psychological readiness in athletic populations. Furthermore, much of the existing sustainability literature is based on general populations rather than athletes and physically active individuals, whose nutritional demands may differ substantially due to training intensity and competition requirements (10).

Therefore, a clearer understanding of the relationship between sustainable dietary behaviors and athlete well-being is needed. Investigating these associations may contribute to the development of sports nutrition approaches that consider not only athletic performance but also long-term health and sustainability. In addition, understanding how athletes perceive the relationship between sustainable nutrition and performance may provide practical implications for coaches, nutritionists, sports organizations, and athlete education programs.

Accordingly, the present study aimed to examine the associations between sustainable food and diet choices, sports psychology outcomes, and perceived physical performance indicators among physically active individuals (11). Specifically, the study investigated whether individuals reporting higher levels of sustainable dietary practices also reported greater motivation, mental focus, mood stability, psychological readiness, energy levels, recovery quality, fatigue control, and perceived physical performance.

The study was guided by the following hypotheses:

*H1*: Sustainable dietary practices are positively associated with sports psychology outcomes among athletes and physically active individuals.

*H2*: Sustainable dietary practices are positively associated with perceived physical performance outcomes.

*H3*: Sustainable dietary practices significantly predict psychological and physical performance outcomes after controlling for demographic and training-related variables.

## 2 Methodology

### 2.1 Research design

This study employed a quantitative cross-sectional research design to examine the associations between sustainable food and diet choices, sports psychology outcomes, and perceived physical performance indicators among physically active individuals. The cross-sectional approach enabled the collection of data from participants at a single point in time using self-reported questionnaire measures.

### 2.2 Study setting

The study was conducted between October 2025 and December 2025 in Guangzhou, China. Participants were recruited from university sports teams, fitness centers, sports clubs, and organized training groups. Data collection was conducted using both online and paper-based questionnaire procedures to improve participant accessibility and response rates.

### 2.3 Participants

The target population consisted of athletes, recreationally active individuals, and sports participants engaged in regular physical training or organized sports activities. Eligible participants included individuals aged 18 years or older who had participated in structured physical training for at least 3 months prior to the study. Individuals with severe injury, diagnosed eating disorders, or medical conditions that could substantially affect normal training participation or dietary behavior were excluded from the study.

A purposive sampling method was used to recruit participants with relevant training and sports experience. Participants were approached through university sports programs, local fitness facilities, amateur sports clubs, and athlete training groups. Sample size estimation was performed using G*Power software version 3.1 for multiple regression analysis. Assuming a medium effect size (*f*^2^ = 0.15), significance level of 0.05, statistical power of 0.80, and five predictor variables, the minimum recommended sample size was 138 participants. To improve statistical reliability and subgroup representation, a larger sample was targeted.

A total of 268 individuals initially responded to the survey. After screening for incomplete responses and missing data, 250 questionnaires were retained for final analysis, resulting in a valid response rate of 93.3%. The independent variable in the study was sustainable dietary practices, which included behaviors related to nutrient-dense food intake, plant-based and local food choices, reduction of highly processed foods, and food waste reduction practices.

The dependent variables included sports psychology outcomes and perceived physical performance outcomes. Sports psychology variables included motivation, mental focus, mood stability, stress management, and psychological readiness. Physical performance variables included perceived energy level, recovery quality, fatigue control, training consistency, endurance, and perceived physical performance. Demographic and training-related variables such as age, gender, sport type, competition level, and training frequency were treated as control variables.

### 2.4 Data collection procedure

Data were collected using both online and paper-based questionnaire methods.

#### 2.4.1 Online data collection

The online questionnaire was developed using Google Forms. The survey link was distributed through university sports networks, social media groups related to sports and fitness, and direct communication with sports clubs and training groups. Participants first viewed an information page describing the purpose of the study, confidentiality procedures, voluntary participation, and estimated completion time. Electronic informed consent was obtained before participants were allowed to access the questionnaire items.

#### 2.4.2 Paper-based data collection

Paper-based questionnaires were administered in person at university sports facilities, training centers, and fitness clubs with prior permission from the respective institutions and coaches. Participants received verbal instructions regarding the purpose of the study and confidentiality of responses before completing the questionnaire. Written informed consent was obtained from all participants prior to participation.

The questionnaire required approximately 10–15 min to complete. Participants were instructed to respond based on their usual dietary habits, training experiences, and perceived psychological and physical performance. No personally identifying information was collected. Completed questionnaires were screened for completeness before data analysis.

### 2.5 Data collection instruments

Data were collected using a structured self-administered questionnaire consisting of four sections.

#### 2.5.1 Demographic questionnaire

The first section collected demographic and training-related information, including age, gender, sport type, competition level, dietary pattern, training frequency, and training duration. These variables were used to describe the sample characteristics and were included as control variables during statistical analysis.

#### 2.5.2 Sustainable nutrition and food choices

The second section assessed sustainable nutrition and food choice behaviors. The items were developed based on existing literature related to sustainable nutrition, sports nutrition, and healthy dietary behaviors. The questionnaire evaluated intake of fruits, vegetables, whole grains, legumes, minimally processed foods, plant-based food choices, local food preferences, and food waste reduction practices.

Responses were measured using a 5-point Likert scale ranging from 1 (“strongly disagree”) to 5 (“strongly agree”), with higher scores indicating greater adherence to sustainable dietary practices. Content validity was evaluated through expert review by specialists in sports nutrition and health sciences. A pilot test involving 20 physically active individuals was conducted to evaluate clarity and readability of the items before full-scale data collection. The sustainable nutrition scale demonstrated satisfactory internal consistency with a Cronbach’s alpha coefficient of 0.84.

#### 2.5.3 Sports psychology outcomes

The third section assessed sports psychology outcomes related to athletic participation. The questionnaire included items measuring motivation, mental focus, mood stability, emotional readiness, confidence, and stress management during training and competition. These items were adapted from commonly used constructs in sports psychology and athlete well-being research.

Responses were rated on the same 5-point Likert scale. Higher scores reflected more positive psychological outcomes and greater psychological readiness for sports participation. The sports psychology section demonstrated strong internal consistency with a Cronbach’s alpha value of 0.88.

#### 2.5.4 Physical performance outcomes

The fourth section evaluated perceived physical performance outcomes. Participants rated their perceived energy levels, recovery quality, fatigue management, endurance, training consistency, and overall physical performance during regular training activities. Items were measured using the same 5-point Likert response format. Higher scores indicated more favorable perceived physical performance outcomes. The physical performance section demonstrated acceptable reliability with a Cronbach’s alpha coefficient of 0.86.

### 2.6 Ethical considerations

The study was conducted in accordance with the ethical principles of the Declaration of Helsinki for research involving human participants. Ethical approval was obtained from the Institutional Research Ethics Committee of Guangzhou Sport University (Approval No.: GSU-2025-SPORT-117; Approval Date: 15 September 2025).

For online participation, electronic informed consent was obtained before participants accessed the questionnaire. For paper-based participation, written informed consent was obtained before questionnaire administration. Participation was voluntary, and participants were informed that they could withdraw from the study at any time without penalty. All responses were collected anonymously and used exclusively for academic and research purposes.

### 2.7 Data analysis

Data analysis was conducted using IBM SPSS Statistics version 27.0. Prior to analysis, the dataset was screened for incomplete responses, missing values, and outliers.

Descriptive statistics including frequency, percentage, mean, and standard deviation were calculated to summarize participant characteristics and study variables. Internal consistency reliability of questionnaire sections was evaluated using Cronbach’s alpha coefficients. Pearson correlation analysis was performed to examine associations between sustainable dietary practices, sports psychology outcomes, and physical performance variables. Multiple linear regression analysis was conducted to determine whether sustainable dietary practices significantly predicted psychological and physical performance outcomes after controlling for demographic and training-related variables. Statistical significance was established at *p* < 0.05.

## 3 Results

### 3.1 Participant characteristics

A total of 268 individuals initially responded to the study questionnaire. Following data screening procedures, 18 responses were excluded because of incomplete questionnaires or missing data patterns exceeding the acceptable threshold (12). Consequently, data from 250 participants were retained for final analysis, resulting in a valid response rate of 93.3%.

**Figure 1:**
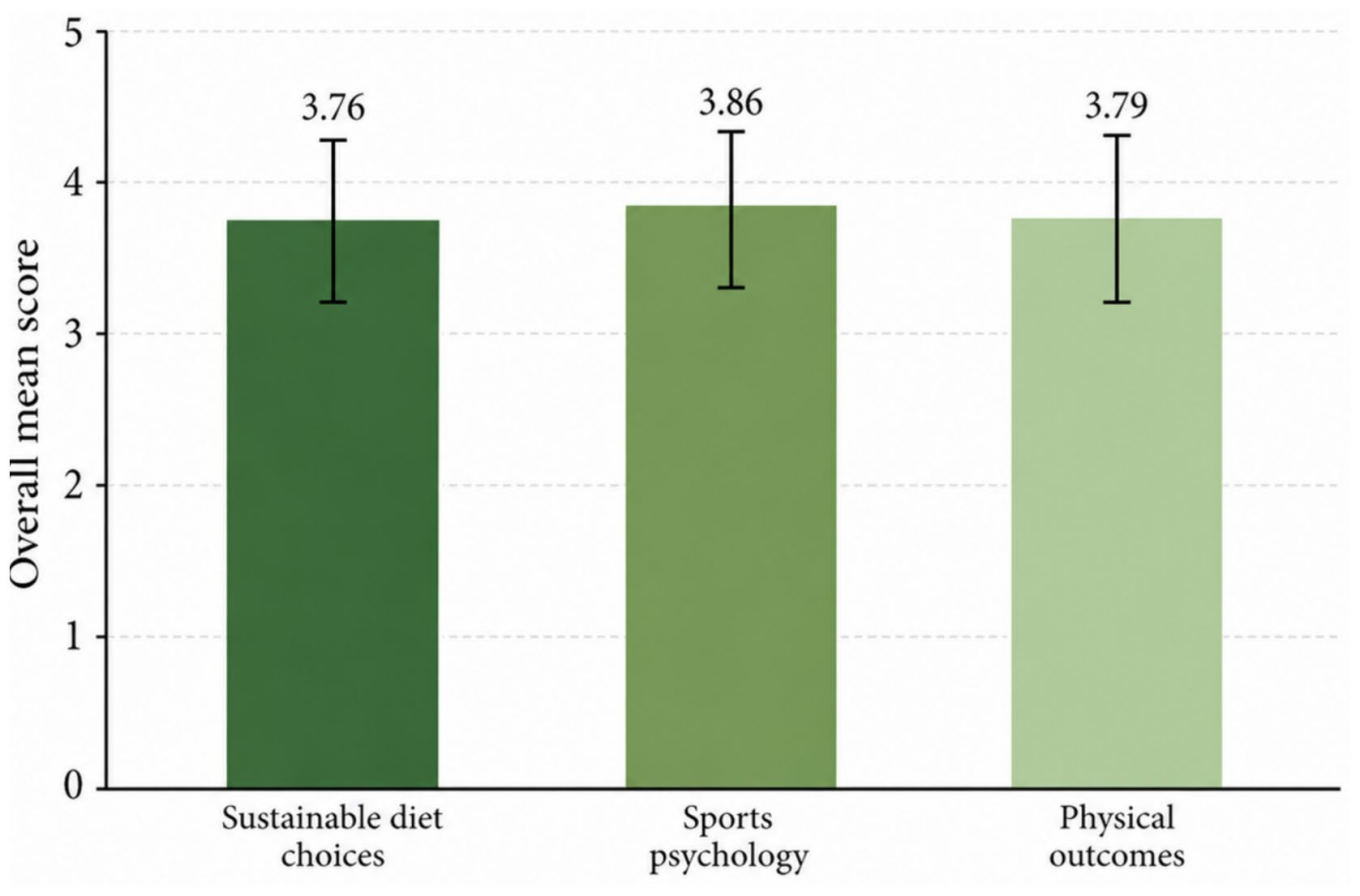
Overall mean scores for sustainable diet choices, sport psychology, and physical outcomes.

The sample included participants from different sporting backgrounds and training levels. Male participants represented 58.4% of the sample, while females accounted for 41.6%. Most participants were between 23 and 27 years of age (40.8%) (13). Team sports participants constituted the largest subgroup (36.4%), followed by endurance sports (25.2%), strength/power sports (20.8%), and recreational fitness participants (17.6%). Regarding training frequency, most respondents reported training 4-5 days per week (51.2%). Mixed dietary patterns were the most commonly reported nutritional approach (55.2%), whereas vegetarian and vegan dietary patterns represented smaller proportions of the sample as shown in Table 1 and Figure 1.

**Table 1:** Demographic and training characteristics of participants.

Variable	Category	Frequency (*n*)	Percentage (%)
Gender	Male	146	58.4
	Female	104	41.6
Age group	18–22 years	78	31.2
	23–27 years	102	40.8
	28–32 years	45	18.0
	Above 32 years	25	10.0
Sport type	Endurance sports	63	25.2
	Strength/power sports	52	20.8
	Team sports	91	36.4
	Recreational fitness	44	17.6
Competition level	Recreational	72	28.8
	University/club level	112	44.8
	Regional/national level	66	26.4
Training frequency	2-3 days/week	53	21.2
	4-5 days/week	128	51.2
	6 or more days/week	69	27.6

### 3.2 Descriptive statistics and reliability analysis

Descriptive statistics were calculated to evaluate sustainable dietary practices, sports psychology outcomes, and perceived physical performance outcomes among participants (14). Overall, respondents reported moderate levels of sustainable dietary behavior and moderately positive psychological and perceived physical outcomes.

Among sustainable nutrition dimensions, sustainable dietary awareness demonstrated the highest mean score (*M* = 3.82, SD = 0.69), while plant-based and local food choices demonstrated comparatively lower scores (*M* = 3.34, SD = 0.81) (15). This finding suggests that although participants were generally aware of sustainable nutrition concepts, the practical implementation of these behaviors may have been more variable.

Within the sports psychology domain, motivation demonstrated the highest mean score (*M* = 3.91, SD = 0.66), whereas mood stability showed relatively lower scores (*M* = 3.52, SD = 0.78). For perceived physical outcomes, energy level scores were relatively higher (*M* = 3.79, SD = 0.71), while fatigue control demonstrated comparatively lower values (*M* = 3.41, SD = 0.80).

Internal consistency analyses indicated acceptable reliability across questionnaire sections (16). Cronbach’s alpha coefficients ranged from 0.81 to 0.86, suggesting satisfactory internal consistency for research purposes.

Exploratory factor analysis was conducted to evaluate construct validity. Kaiser-Meyer-Olkin values exceeded 0.70, and Bartlett’s test of sphericity was statistically significant (*p* < 0.001), supporting the suitability of the data for factor analysis as presented in Table 2.

**Table 2:** Descriptive statistics and reliability of study variables.

Variable/dimension	Mean	SD	Cronbach’s alpha
Sustainable dietary awareness	3.82	0.69	
Nutrient-dense food choices	3.71	0.72	
Plant-based/local food choices	3.34	0.81	
Food waste reduction behavior	3.49	0.77	
Overall sustainable dietary practices	3.59	0.58	0.84
Motivation	3.91	0.66	
Mental focus	3.63	0.71	
Mood stability	3.52	0.78	
Psychological readiness	3.69	0.70	
Overall sports psychology outcomes	3.69	0.56	0.86
Energy level	3.79	0.71	
Recovery quality	3.48	0.76	
Fatigue control	3.41	0.80	
Perceived physical performance	3.57	0.73	
Overall perceived physical outcomes	3.56	0.59	0.81

### 3.3 Preliminary assumption testing

Prior to inferential analyses, statistical assumptions were evaluated. Normality assessment using skewness and kurtosis statistics indicated acceptable distributions for all major variables, with values remaining within recommended ranges (±2). Scatterplot examination suggested no major violations of linearity or homoscedasticity assumptions (17).

Multicollinearity diagnostics indicated acceptable tolerance values (>0.20) and variance inflation factor (VIF) values ranging from 1.18 to 2.06, suggesting no substantial multicollinearity concerns among predictor variables. Residual analyses also indicated that the assumptions required for multiple regression analysis were adequately met (18).

### 3.4 Correlation analysis

Pearson correlation analysis was conducted to examine associations between sustainable dietary practices, sports psychology outcomes, and perceived physical outcomes.

**Figure 2:**
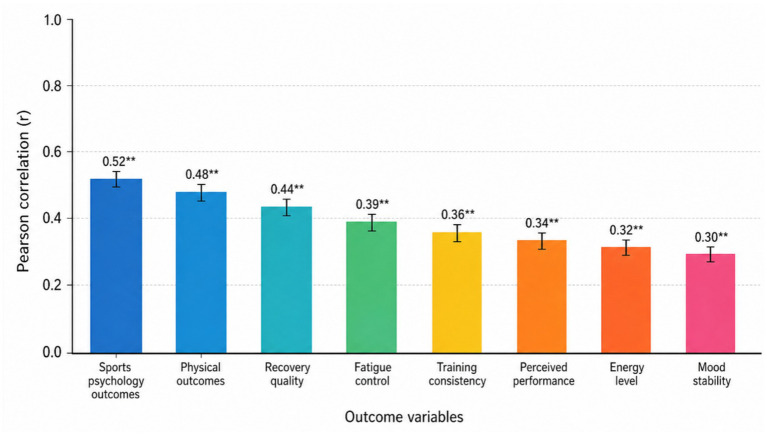
Correlation between sustainable diet choices and key psychological and physical outcomes among athletes.

Sustainable dietary practices demonstrated a moderate positive association with sports psychology outcomes (*r* = 0.41, *p* < 0.001) (19). This suggests that participants reporting healthier and more sustainability-oriented dietary behaviors also tended to report more favorable motivation, focus, and psychological readiness as shown in Figure 2.

A weaker but statistically significant association was observed between sustainable dietary practices and perceived physical outcomes (*r* = 0.34, *p* < 0.001) (20). Recovery quality also demonstrated a positive relationship with sustainable dietary practices (*r* = 0.29, *p* < 0.01). In contrast, the relationship between sustainable dietary practices and training consistency was relatively weak and did not reach statistical significance (*r* = 0.11, *p* = 0.087) (21).

**Figure 3:**
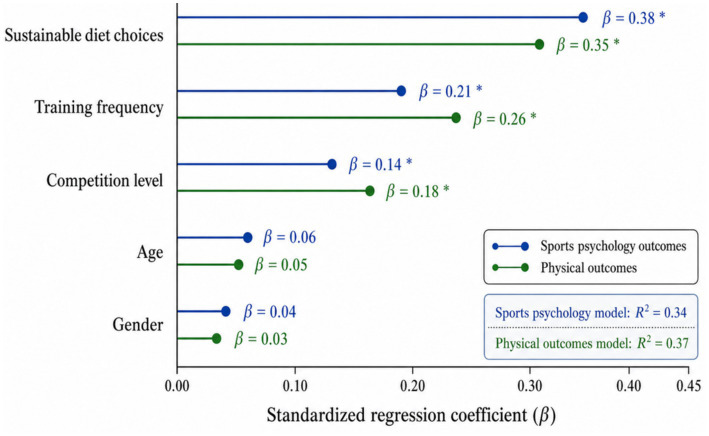
Standardized regression coefficients showing the predictive effect of sustainable diet choices and training-related variables on sports psychology and physical outcomes among study participants.

Sports psychology outcomes demonstrated moderate positive relationships with perceived physical outcomes (*r* = 0.49, *p* < 0.001), suggesting that psychological readiness and perceived physical functioning may be interconnected within athletic settings as shown in Figure 3 and Table 3.

**Table 3:** Correlation matrix among major study variables.

Variable	1	2	3	4	5	6
1. Sustainable dietary practices	1					
2. Sports psychology outcomes	0.41**	1				
3. Perceived physical outcomes	0.34**	0.49**	1			
4. Recovery quality	0.29**	0.38**	0.52**	1		
5. Fatigue control	0.18*	0.31**	0.47**	0.44**	1	
6. Training consistency	0.11	0.26**	0.33**	0.21*	0.19*	1

Values represent pearson correlation coefficients (r). *indicates *p* < 0.05, and **indicates *p* < 0.01. Correlations without asterisks are not statistically significant.

### 3.5 Regression analysis

Multiple linear regression analyses were conducted to examine whether sustainable dietary practices significantly predicted sports psychology outcomes and perceived physical outcomes after controlling for demographic and training-related variables.

In Model 1, sustainable dietary practices significantly predicted sports psychology outcomes (*β* = 0.31, *p* < 0.001) (22). Training frequency also demonstrated a statistically significant contribution (*β* = 0.19, *p* = 0.004), whereas age and gender were not significant predictors. The overall model explained 24% of the variance in sports psychology outcomes (Adjusted *R*^2^ = 0.22).

In Model 2, sustainable dietary practices significantly predicted perceived physical outcomes (*β* = 0.27, *p* < 0.001). Training frequency and competition level also contributed significantly to the model (23). However, the overall explanatory power of the model remained moderate (Adjusted *R*^2^ = 0.25), indicating that additional factors not measured in this study may also influence perceived physical outcomes as shown in Table 4.

**Table 4:** Multiple regression analysis predicting psychological and perceived physical outcomes.

Model	Dependent variable	Predictor	*β*	*t*	*p*	5% CI
Model 1	Sports psychology outcomes	Sustainable dietary practices	0.31	4.82	<0.001	[0.18, 0.44]
		Training frequency	0.19	2.94	0.004	[0.06, 0.31]
		Competition level	0.10	1.76	0.080	[−0.01, 0.21]
		Age	0.05	0.88	0.382	[−0.07, 0.16]
		Gender	0.03	0.54	0.592	[−0.08, 0.14]
		Model statistics	Adjusted *R*^2^ = 0.22	*F* = 13.74	<0.001	
Model 2	Perceived physical outcomes	Sustainable dietary practices	0.27	4.19	<0.001	[0.14, 0.39]
		Training frequency	0.22	3.26	0.001	[0.09, 0.35]
		Competition level	0.16	2.41	0.017	[0.03, 0.28]
		Age	0.04	0.73	0.468	[−0.08, 0.15]
		Gender	0.02	0.41	0.684	[−0.09, 0.12]
		Model Statistics	Adjusted *R*^2^ = 0.25	*F* = 15.06	<0.001	

**Figure 4:**
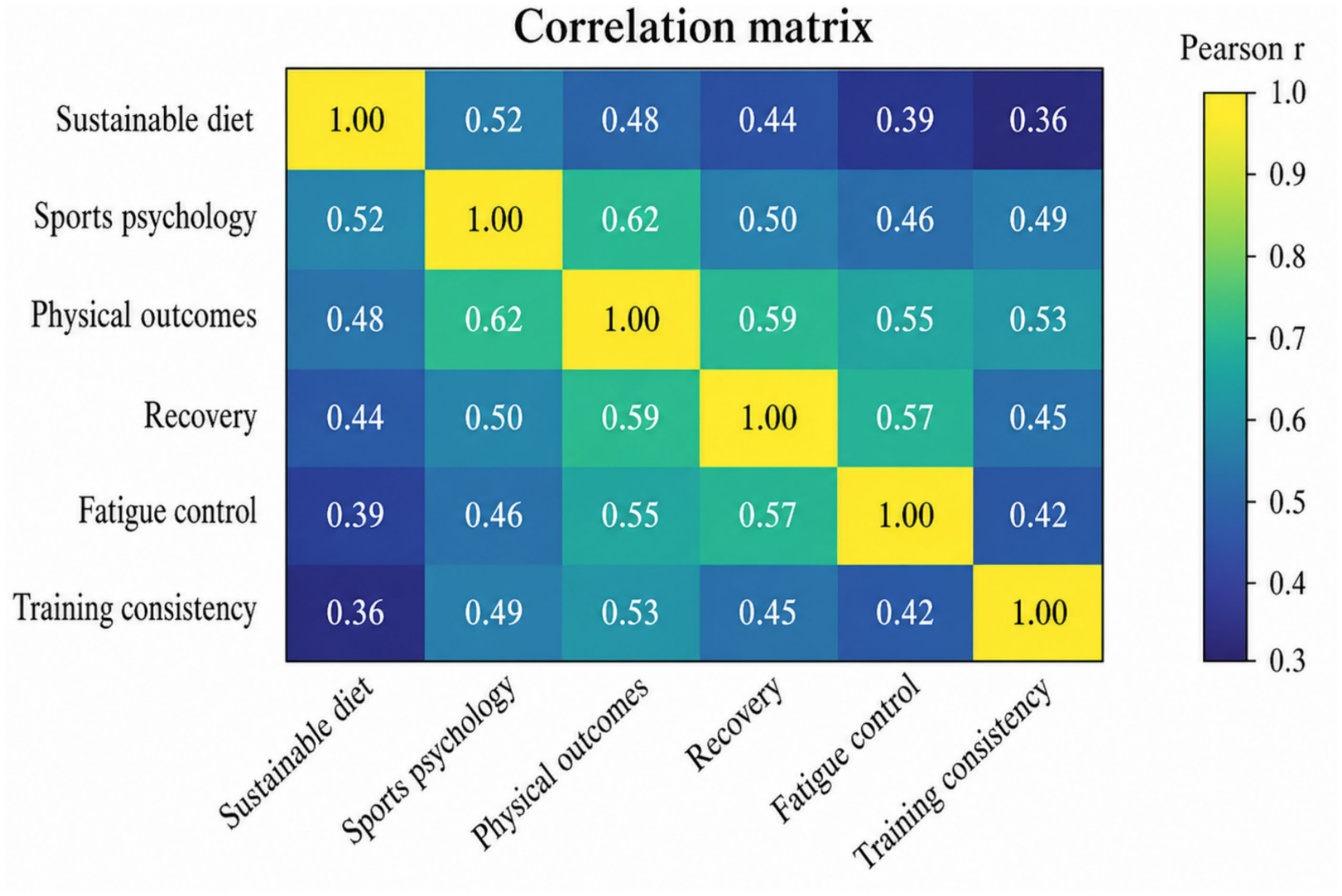
Pearson correlation matrix showing the relationship among sustainable diet choices, sport psychology, physical outcomes, recovery, fatigue control and training consistency.

Figure 4 presents the Pearson correlation matrix among all major study variables. Sustainable diet demonstrated moderate positive correlations with sports psychology (*r* = 0.52), physical outcomes (*r* = 0.48), recovery (*r* = 0.44), fatigue control (*r* = 0.39), and training consistency (*r* = 0.36), indicating that higher dietary adherence was broadly associated with more favorable outcomes across all domains. The strongest association in the matrix was observed between sports psychology and physical outcomes (*r* = 0.62), suggesting that psychological and physical functioning are more closely interrelated with each other than either is with dietary behavior alone. Recovery showed notable correlations with both physical outcomes (*r* = 0.59) and fatigue control (*r* = 0.57), reflecting the interconnected nature of these performance-related variables. Training consistency demonstrated the weakest associations across the matrix, particularly with sustainable diet (*r* = 0.36), consistent with the regression findings that training adherence is influenced by factors beyond dietary practices.

**Figure 5:**
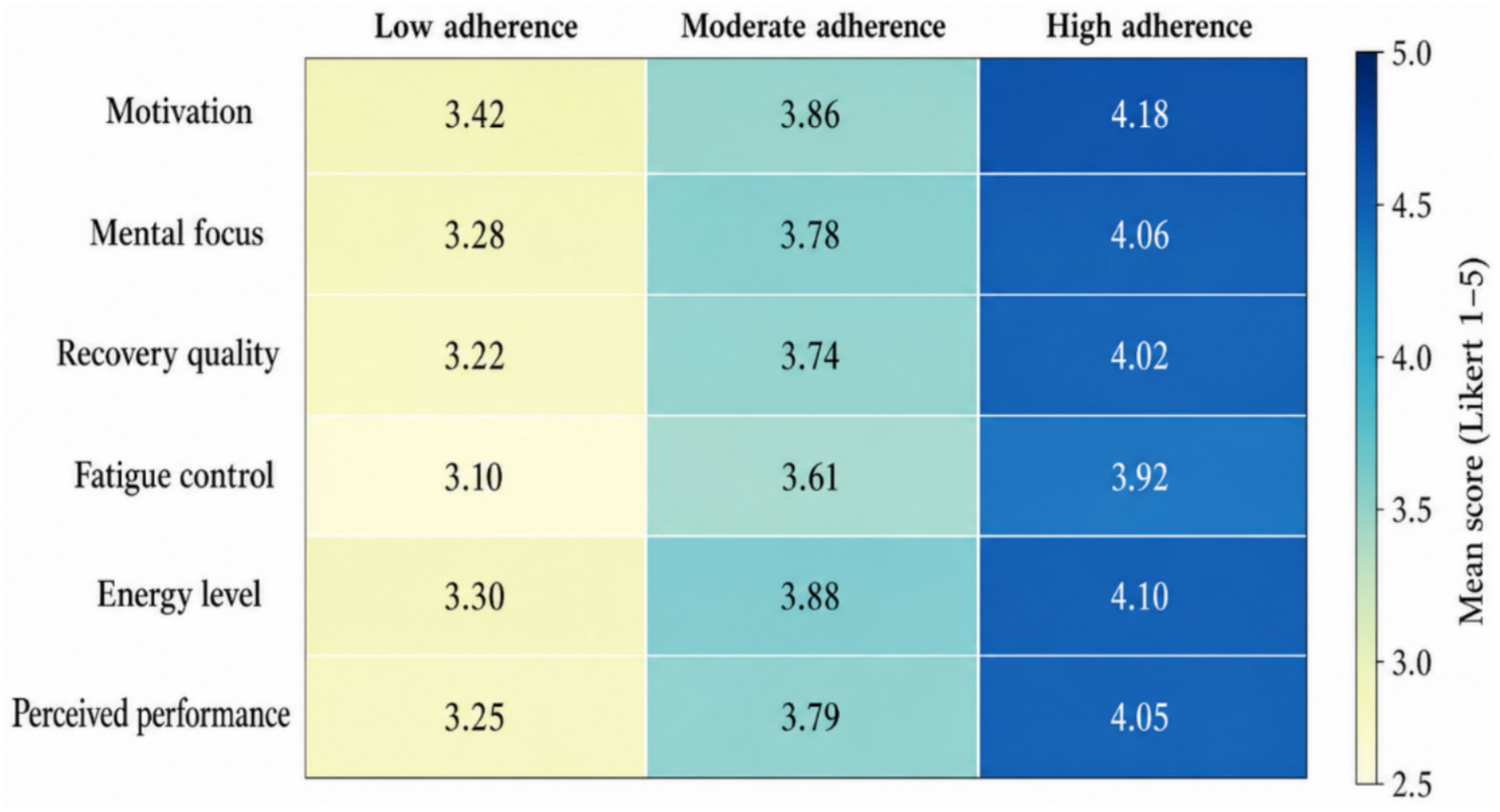
Heatmap showing higher psychological and physical scores among athletes with greater adherence to sustainable diet practices.

Figure 5 illustrates that mean scores across all psychological and physical outcome dimensions increased progressively from low to high adherence to sustainable dietary practices. Motivation recorded the highest scores across all adherence groups (3.42, 3.86, and 4.18 for low, moderate, and high adherence, respectively), while fatigue control consistently showed the lowest values (3.10, 3.61, and 3.92). Participants with high adherence scored notably higher on energy level (4.10) and perceived performance (4.05) compared to those with low adherence (3.30 and 3.25, respectively), suggesting a meaningful gradient between dietary adherence and perceived physical functioning. The pattern was consistent across both psychological and physical domains, with no outcome dimension showing a reversal in trend across the three adherence groups. These results visually support the positive associations identified in the correlation and regression analyses, although the cross-sectional and self-reported nature of the data precludes causal interpretation.

## 4 Discussion

The present study examined the associations between sustainable dietary practices, sports psychology outcomes, and perceived physical outcomes among physically active individuals (24). Overall, the findings suggest that participants reporting healthier and more sustainability-oriented dietary behaviors also tended to report more favorable psychological well-being and perceived physical functioning (25). However, the observed relationships were generally moderate rather than strong, indicating that dietary practices likely represent only one of several factors influencing athlete well-being and perceived performance.

One of the primary findings of the study was the moderate positive association between sustainable dietary practices and sports psychology outcomes (26). Participants who reported greater adherence to nutrient-dense and sustainability-oriented dietary behaviors also tended to report higher levels of motivation, mental focus, and psychological readiness as shown in Figure 4. These findings are consistent with sports nutrition literature suggesting that dietary quality may contribute to psychological functioning through mechanisms related to stable energy availability, micronutrient adequacy, inflammation regulation, and overall health status (27). Nutritional factors may influence neurotransmitter synthesis, cognitive functioning, and mood regulation, which are important components of athletic preparation and psychological resilience.

The findings also demonstrated positive associations between sustainable dietary practices and perceived physical outcomes, including recovery quality and energy levels (28). Nutrient-dense dietary patterns rich in fruits, vegetables, whole grains, legumes, nuts, and minimally processed foods may contribute to recovery processes through antioxidant activity, glycogen restoration, and reduced inflammatory responses following exercise. Nevertheless, the relationships observed in this study were relatively moderate, suggesting that perceived physical functioning is influenced by multiple interacting factors such as sleep quality, training load, coaching strategies, injury status, and recovery practices (29).

An important finding was that the relationship between sustainable dietary practices and training consistency was relatively weak and statistically nonsignificant. This result suggests that training adherence may depend more heavily on external and behavioral factors beyond nutrition alone, including competition schedules, access to training facilities, motivational climate, social support, and time management demands (30). The presence of weaker and nonsignificant relationships in some analyses further indicates that the associations identified in this study should not be interpreted as universally strong or deterministic.

Regression analyses demonstrated that sustainable dietary practices remained statistically significant predictors of sports psychology outcomes and perceived physical outcomes even after controlling for demographic and training-related variables (31). However, the explained variance of the models remained moderate, indicating that substantial variability in psychological and perceived physical functioning remains unexplained. This finding is expected within behavioral and sports science research, where performance-related outcomes are influenced by complex physiological, psychological, social, and environmental interactions (32).

The present findings contribute to the growing literature on sustainable sports nutrition by extending existing research beyond environmental sustainability and traditional physiological performance outcomes. Previous studies have primarily focused on macronutrient intake, supplementation practices, or environmental sustainability independently. In contrast, the current study attempted to examine sustainable dietary behaviors alongside both psychological and perceived physical dimensions within an athletic context (33). Nevertheless, the findings should be interpreted cautiously due to the self-reported nature of the measures and the cross-sectional study design.

Several practical implications emerge from the findings. Sports nutrition professionals and coaches may benefit from considering sustainable dietary practices not solely from an environmental perspective but also within broader athlete well-being frameworks. Encouraging balanced and nutritionally adequate dietary patterns that emphasize minimally processed foods and dietary variety may contribute positively to athlete health and perceived readiness (34). However, sustainable dietary approaches should remain individualized according to sport type, training demands, cultural preferences, and nutritional requirements.

Despite these contributions, the study has important limitations. The cross-sectional design prevents causal inference regarding the relationships identified. In addition, all variables were measured using self-report questionnaires, which may introduce recall bias, social desirability bias, and common method variance. The study also relied on perceived rather than objective measures of physical performance (35). Future research should incorporate objective physiological and performance indicators, longitudinal designs, and more diverse athlete populations to further examine the relationship between sustainable dietary practices and athlete well-being.

## 5 Limitations

This study has several limitations that should be considered when interpreting the findings. First, the cross-sectional design prevents conclusions regarding causal relationships between sustainable dietary practices and athlete outcomes. Second, all measures were based on self-reported perceptions, which may be influenced by recall bias and social desirability effects. Third, perceived physical outcomes were evaluated rather than objective laboratory or field-based performance indicators. Fourth, the purposive sampling strategy may limit the generalizability of findings to broader athlete populations. Finally, although reliability and preliminary validity analyses supported the questionnaire structure, further psychometric validation in larger and more diverse athletic samples is recommended.

## 6 Conclusion

This study examined the associations between sustainable dietary practices, sports psychology outcomes, and perceived physical outcomes among physically active individuals. The findings consistently indicated that sustainable dietary practices were positively and significantly associated with both sports psychology and perceived physical outcomes. Regression analyses further confirmed that sustainable dietary practices remained a meaningful and independent predictor of both outcome domains after accounting for training frequency, competition level, age, and gender. Among the covariates considered, training frequency also emerged as a significant contributor to both models, whereas age and gender did not demonstrate notable predictive value. The models collectively accounted for a moderate proportion of variance in the outcome measures, which is consistent with the multifactorial nature of athletic performance and well-being, and underscores the likelihood that additional psychological, physiological, social, and environmental factors play important roles beyond those captured in the present study.

Examination of sub-dimensions revealed that sustainable dietary awareness was the most strongly endorsed component among participants, whereas plant-based and locally sourced food choices reflected comparatively lower levels of adherence, suggesting these areas represent meaningful targets for future intervention. Within the psychological domain, motivation emerged as the highest-rated outcome, while mood stability was comparatively lower, pointing to nuanced relationships between specific dietary behaviors and distinct psychological states. Regarding perceived physical outcomes, energy level received the most favorable ratings, while fatigue control was relatively less endorsed. Notably, the association between sustainable dietary practices and training consistency was weak and did not attain significance, suggesting that adherence to training regimens is more strongly governed by factors beyond dietary behavior alone. Taken together, these findings highlight the relevance of sustainable nutrition within sport and exercise contexts, while also acknowledging the complexity of performance-related outcomes. Future longitudinal and experimental research is warranted to establish causal pathways and to identify the mechanisms through which dietary choices may shape both the psychological and physical dimensions of athletic performance.

## Data Availability

The raw data supporting the conclusions of this article will be made available by the authors, without undue reservation.
